# Case of Tetanic Affection, in Which the Oleum Terebinthinæ Was Successfully Employed

**Published:** 1823-05

**Authors:** William Toms

**Affiliations:** Member of the Royal College of Surgeons.


ART. V.—*Case of Tetanic Affection, in which the Oleum Terebinthinæ was successfully employed.* By WILLIAM TOMS, Esq. Member of the Royal College of Surgeons.

HAVING lately read the report of a case of tetanus cured by the oleum terebinthinæ, recorded in a late Number of your valuable Journal, I am induced to trouble you with this communication.

Susanna Somerton, a young woman, residing in this town, about twenty-five years of age, had been for a considerable time past the subject of attacks of epilepsy, the exciting cause of which was attributed to fright. On being called to attend her, I perceived the energies of the brain and her intellectual powers had been much enfeebled, which I supposed was caused by the severe paroxysms of epilepsy she was subject to. Scarcely a week passed on without her being attacked with the most violent convulsions; after they had subsided, she remained in a state of stupor for a considerable time, and appeared perfectly insensible to what had taken place. The patient being of a full plethoric habit of body, and considering the symptoms indicated a determination of blood to the head, she was bled freely from the arm. I then applied blisters to the head and neck; she was also freely purged with the Pulv. Scam. cum Cal. and Infus. Sennæ, cum Magn. Sulph. I then followed up the treatment by giving her some of the most powerful antispasmodics. She was evidently relieved by this treatment, the paroxysms being less frequent, and much less severe.

Her mother called here early one morning, to inform me this young woman was again suffering from a severe attack of those fits; and they were dreadfully alarmed by her being unable to open her jaws, which had remained immovably closed for eleven hours. On visiting the patient, I found her in the state described by her mother. She had complained of great pain and stiffness in the back part of the head; also at the lower part of the sternum, shooting to the back; the muscles of the spine were much affected, and the trunk of the body forcibly bent backwards; the extremities rigidly extended, and the stiffened parts affected with violent contractions; the eyes immovable in the sockets, and considerably protruded; pupils dilated; her countenance denoted intense anxiety; and the pulse 110. Fortunately she had two decayed teeth; therefore, I repeated my former treatment with little difficulty. Two blisters were also applied on either side the jaws. In two days the jaws opened, and she was enabled to use her mouth as before.

Five days after, she was attacked in a similar manner, and the jaws remained closed for three days. The same treatment was adopted, with equal good success. She continued tolerably well for eleven hours, when all the former symptoms returned with redoubled vigour. The same plan of treatment was pursued, without the slightest effect, and every thing that was administered proved ineffectual. Her jaws remained locked for six days and nights, when I received the London Medical and Physical Journal, and was much pleased to find a case of tetanus cured by the oleum terebinthinæ. I therefore gave my patient immediately Ol. Terebinth. ℥ss. in a little gruel, and repeated it every three hours. The third dose completely restored her; the mouth opened, and she regained the proper command over the muscles. The spasms were much relieved on taking the second dose; and, on repeating the third, the bowels became excessively purged, and violent vomiting was produced. She also described a peculiar sensation felt at the extreme points of the finger and toes, after taking the turpentine. The jaws have been locked four times since the first exhibition of the ol. terebinthinæ, and each time it was repeated with equally good success. The mouth opened within one hour after the second dose was given. The patient's health is generally improved.

However trifling may be the observations I have troubled you with, I well know you will pardon me for presuming to submit them to your notice. They are in themselves of little or no value; but they may serve as hints for the more successful treatment of an affection which has so frequently defied the most zealous efforts of the surgeon.

***Kingsbridge, Devon; March* 2, 1823.**

